# Role of T-lymphocytes in Kidney Disease

**DOI:** 10.7759/cureus.19153

**Published:** 2021-10-30

**Authors:** Purva Bavikar, Tushar Dighe, Pavan Wakhare, Nilesh Shinde, Charan Bale, Atul Sajgure

**Affiliations:** 1 Nephrology, Dr D Y Patil Hospital, Medical College and Research Institute, Pune, IND

**Keywords:** chronic kidney disease, acute kidney injury, t-regulatory cells, foxp3, ror gamma t

## Abstract

Background

The role of T-regulatory cells (Tregs) in inflammatory renal disease is not yet established. We attempted to study peripherally circulating T-cells expressing ROR*γ*t^+^Foxp3^+^ dynamics in acute kidney injury (AKI) and chronic kidney disease (CKD).

Aim

To determine the role of T-regulatory cells in AKI and CKD.

Research methodology

This is a cross-sectional study conducted between January 2019 to January 2021 at a single tertiary care centre in Pune, India. Candidates enrolled in the study were either patients with CKD not on maintenance hemodialysis or newly diagnosed cases of AKI. Kidney transplant recipients, patients with autoimmune diseases like systemic lupus erythematosus (SLE), IgA nephropathy, or those receiving immuno-suppressants were excluded. T-lymphocytes were analyzed using a flow cytometer.

Results

We studied 80 patients with kidney injury, 40 each belonging to the AKI and CKD study groups and 10 healthy volunteers as controls. The rationale behind having a small control group was to merely get an idea of T helper 17 (Th17):Treg ratio and different immune cell-phenotype profiles in healthy volunteers without kidney injury, diabetes, hypertension or any other risk factors. The ratio of ROR_γ_t:Foxp3 was ≤ 1 in these individuals (control group) while this ratio was significantly altered (MFI ROR_γ_t:Foxp3 ≥1) in the AKI/CKD study arm. We examined peripherally circulating T-lymphocytes in acute kidney injury and chronic kidney disease, comparing their activity to healthy volunteers. Biopsy-proven kidney injury patients (29/80) were also included in this study. We found that the ratio of RORγt:Foxp3 was altered in patients with kidney injury (acute and chronic) and was statistically significant compared to controls, indicating that injury may be attributed to T-cell dysfunction.

Conclusion

Our study provides some evidence of T-cell dysfunction in the pathology of kidney injury in acute and chronic kidney disease via activity of Foxp3 and RORγt. We found that there is evidence of altered Th17/Treg activity in kidney injury, more prevalent in acute than chronic, when compared to healthy volunteers.

## Introduction

The kidney is a highly vascular organ, vital for maintaining internal homeostasis via removal of toxins from the blood. Its anatomical structure and function render it vulnerable to a variety of immune, and non-immune mediated injury.

Kidney disease is a heterogeneous group of disorders affecting its structure and/or function. Inflammation contributes to a myriad of acute and chronic diseases affecting the kidneys. Duration of more than three months is defined as chronic, while duration of three months or less is termed as acute. There is a complex relationship between acute kidney injury (AKI) and chronic kidney disease (CKD); AKI can lead to CKD, and CKD increases the risk of AKI [1].

The immune system is designed to guard the body against pathogens and other harmful signals. T-regulatory cells (Tregs) play a crucial role in several inflammatory phenomena, including those in the kidney [2]. Tregs play a pivotal role in maintaining immune homeostasis by preventing its abnormal activation throughout the lifespan thus preventing autoimmune disease, including those occurring in the kidney [2]. The fork-head box protein 3 transcription factor (Foxp3)-expressing CD4+ Tregs make up a subset of the helper T cells (Th). Peripheral tolerance is attributed to Tregs/helper T cells, which are derived from the thymus or generated peripherally.

Bi-functional Tregs (biTregs) are cells that express both Th17 transcription factor retineic-acid-receptor-related orphan nuclear receptor gamma (RORγt) and Treg-inducing transcription factor Foxp3 [3]. Their role in inflammatory renal disease is not yet established. Ivanov et al. iterate administration of Foxp3+ Tregs had a protective role in glomerulonephritis while depletion of Foxp3+ Tregs was shown to greatly aggravate renal injury. Contrarily, the most potent master transcription factor for inducing pathogenic Th17 responses is RORγt [4]. In fact, central pro-inflammatory mediators of glomerulonephritis are RORγt and its target cytokine IL-17 [5-8].

It is thus fascinating to hypothesize that cells expressing both of these transcription factors, Foxp3 and RORγt, play an important role in inflammatory kidney disease. Thus given the clinical implications, it was decided to study peripherally circulating T-cells expressing RORγt+Foxp3+ dynamics in AKI and CKD.

## Materials and methods

Our study was a single-center, cross-sectional study conducted between January 2019 to January 2021. This study was in accordance with the principles of the Declaration of Helsinki and good clinical practice, along with the institutional regulatory guidelines. After seeking approval from the institutional ethical committee of Dr D.Y Patil Vidyapeeth, Pune (approval IESC/S.SP/2018/08), patients who presented with acute kidney injury or chronic kidney disease, either to the out-patient department or hospitalised in the ward or critical care unit of Dr D.Y Patil Hospital, Pune, were studied. The study population was divided into three arms, healthy volunteers, AKI and CKD. Patients aged 18-65 years, both male and female were evaluated and categorized according to the aforementioned study arms. AKI and CKD were defined by the AKIN (Acute Kidney Injury Network) criteria [9] and KDOQI (Kidney Disease Outcomes Quality Initiative) [10] respectively. Patients belonging to the CKD arm were further divided into five stages based on estimated glomerular filtration rate (eGFR) according to 2012 KDIGO (Kidney Disease Improving Global Outcomes) guidelines [11].

We excluded kidney transplant recipients, patients with autoimmune kidney disease or those taking steroids or other immunosuppressive agents. HIV, hepatitis B surface antigen (HBsAg) or hepatitis C virus (HCV)-positive candidates were also not included in our study. None of the candidates included in the study had active tuberculosis. Patients with solitary kidney (congenitally, renal-agenesis or post-nephrectomy, etc) and with AKI having bilateral kidney size < 8cm in longitudinal axis as seen on sonography were also dropped out.

**Figure 1:**
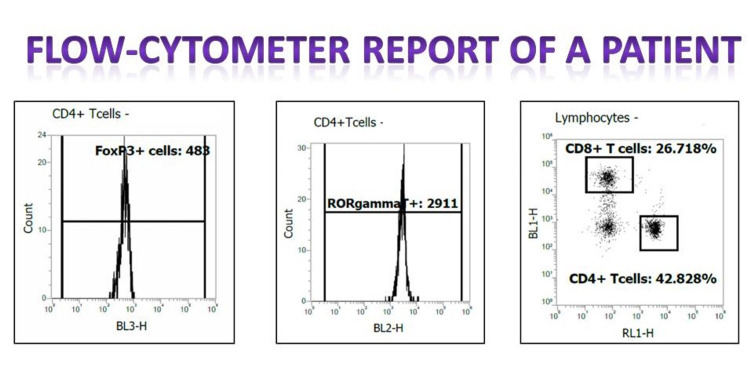
T-cell report depicting FOXp3+ and ROR Gamma T activity in a patient with kidney injury. (Image modified to fit publication requirements)

Individuals who did not give written consent to participate in the study were debarred. All candidates underwent ultrasonography of the abdomen, routine blood and urine investigations like hemogram, serum urea, creatinine, HbA1c, dipstick urine routine, microscopy, urine protein creatinine ratio (UPCR). A 3ml sample of blood collected in EDTA bulb was subjected to Ficoll gradient method for making a single cell preparation of peripheral blood mononuclear cells (PBMCs). After extracting T-lymphocytes, they were analysed with a flow cytometer with appropriate instrument setting. Reports from the flow cytometer were received in the format given in Figure 1. 

Clinical data from the case record proforma which included medical history, physical examination, laboratory and radiological investigations were compiled on MS Office Excel Sheet (v2019, Microsoft, Redmond, WA, USA) and was subjected to statistical analysis using Statistical Package for Social Sciences (SPSS) version 26 (IBM Corp., Armonk, NY, USA). Inter-group comparison of data (two groups) was done using t-test, whereas comparison of more than two groups was done using ANOVA followed by pair-wise comparison using post hoc test. Chi square test was used for comparison of frequencies of categories of variables with groups. For all the statistical tests, p<0.05 was considered to be statistically significant, keeping α error at 5% and β error at 20%, thus giving a power to the study as 80%. A p<0.01 was interpreted as statistically highly significant whereas p>0.05 was not significant.

## Results

We studied 80 patients with kidney injury, 40 with acute kidney injury and 40 patients with chronic kidney disease. Ten healthy volunteers were included as controls. The rationale behind having a small size of control group was to gain clarity regarding T-cell activity in health i.e to get an idea of Th17:Treg via RORγt:Foxp3 activity and T-cell phenotypes profile in individuals without diabetes, hypertension, pre-existing kidney injury or any other risk factor or health ailment. The ratio of mean fluorescence intensity (MFI) RORγT:FoxP3 activity was found to be ≤1 in the controls of our study population whereas this ratio was ≥1 in the kidney injury group. This helped us strengthen our hypothesis. 

**Figure 2:**
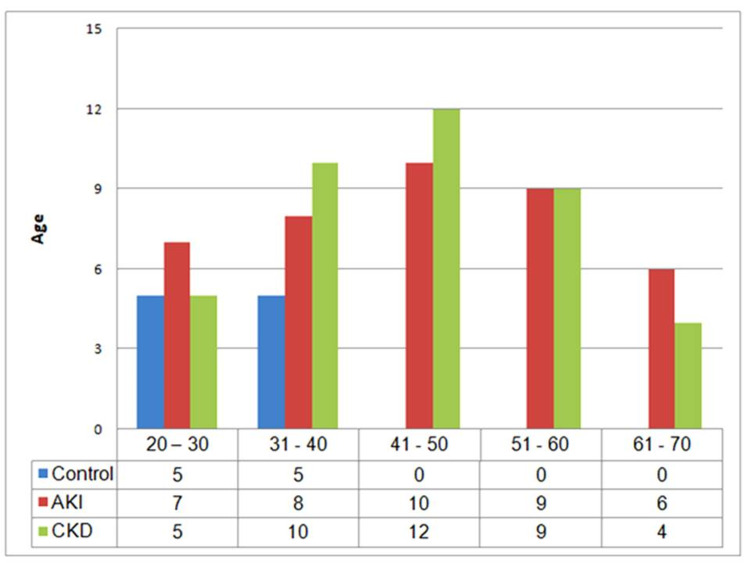
Age distribution of the study population. Healthy volunteers belonged to two age groups i.e 20 - 30 years and 31 - 40 years. Patients with AKI and CKD were distributed in five sets of age groups i.e 20 - 30 years, 31 - 40 years, 41- 50 years, 51 - 60 years and 61 - 70 years. AKI = Acute Kidney Injury; CKD = Chronic Kidney Disease

A total of 60 men and 20 women with kidney injury were evaluated. The age distribution of the study population is depicted in Figure 2.

**Figure 3:**
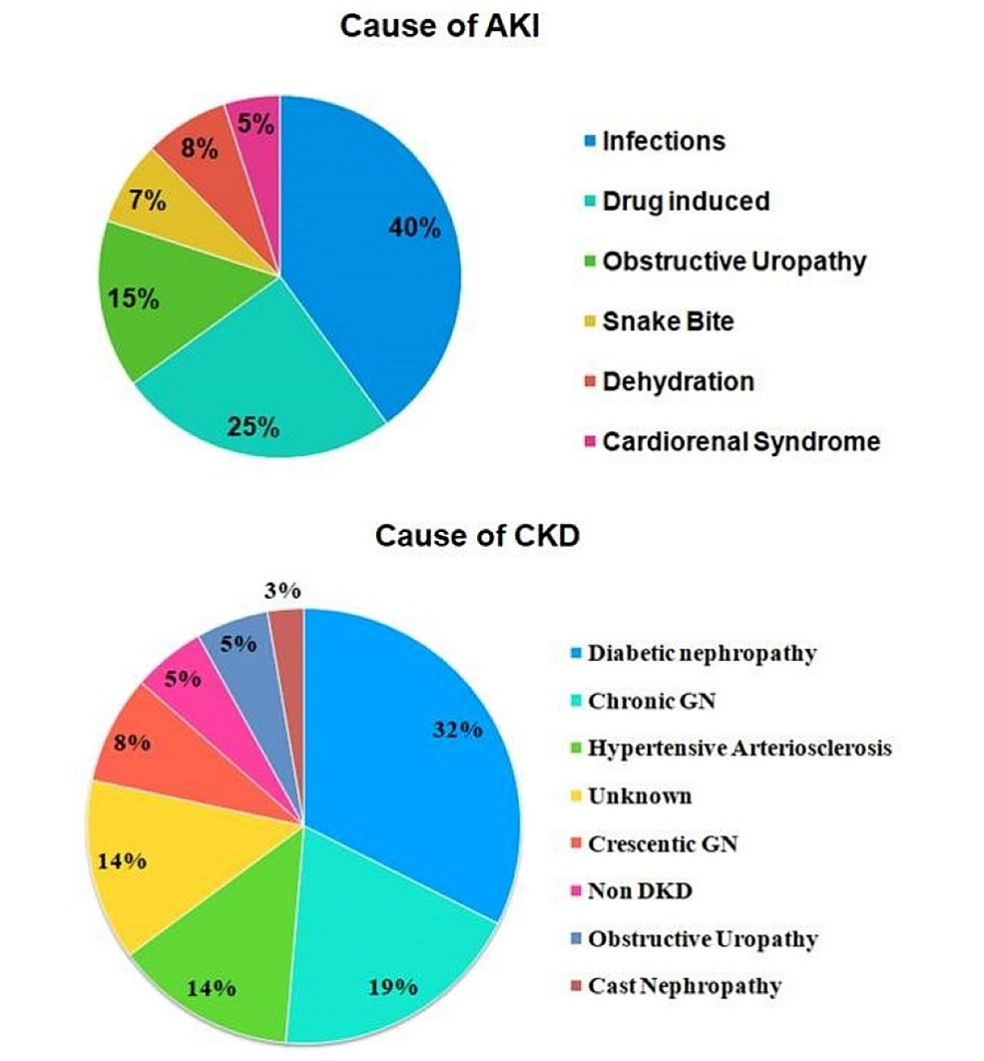
Etiology of AKI & CKD in study population AKI = Acute Kidney Injury; CKD = Chronic Kidney Disease

27.5% of patients with both acute and chronic kidney injury were in their fourth decade of life, whereas 15% of patients were between 20 - 30 years and 12.5% of patients belonged to the 61-70 years age group. Fifty-two percent of the study population had hypertension. Nineteen of 40 patients with AKI and 28 of 40 patientswith CKD included in the study were hypertensive. Thirty percent of the patients included in the study were diabetics. Figure 3 illustrates causes of AKI and CKD in the study population.

AKI study arm

Infections, especially urinary tract infections (UTIs) and sepsis, were the major cause of AKI in our study population (40%), followed by drug induced nephropathy. Fifteen percent of patients in the AKI study arm had kidney injury due to nephrolithiasis with obstructive uropathy. The other causes of AKI in our study were snake bite, dehydration and cardio renal syndrome. 

Seventy percent of patients with AKI belonged to stage 3 of the AKIN criteria whereas only 15% of patients with AKI belonged to stage 1 and 2 each.

CKD study arm

Thirty-two percent of the CKD study arm had diabetic nephropathy as the primary cause of chronic injury followed by chronic glomerulonephritis. Other causes of CKD were long standing obstructive uropathy, non diabetic kidney disease, crescentic glomerulonephritis, cast nephropathy, and CKD of unknown origin.

Based on eGFR (CKD-EPI), 2012 KDIGO classification, CKD patients were distributed into five stages, of which the maximum (52.5%) patients belonged to CKD stage IV (G4). 32.5% were found to have CKD-V (G5) and 15% had CKD IIIb (G3b). Incidentally, no patients in the study belonged to CKD stage G1, G2, or G3a.

Significance of RORγt and Foxp3 CD4 cells in kidney injury

RORγt is one of the master regulators in the development of Th17 cells. Dellepiane et al. demonstrated that Th17 are the most abundant kidney infiltrating-lymphocytes following AKI in mice. In our study, MFI RORγt values in the AKI study arm when compared to healthy volunteers and CKD patients were found to be statistically significant (p<0.01). Similarly the ratio of RORγt:Foxp3 was also statistically significant for the AKI study arm, as seen in Table 1. 

**Table 1:** Significance of RORγt+Foxp3+ in healthy controls and kidney disease (AKI & CKD) Abbreviations used in Table 1: MFI = Mean fluorescence intensity; RORγt+ = Retineic-acid-receptor-related orphan nuclear receptor gamma; Foxp3 = fork-head box protein 3; AKI = Acute Kidney Injury; CKD = Chronic Kidney Disease Annotations for Table 1: * = statistically significant difference (p<0.05) ** = statistically highly significant difference (p<0.01) # = non significant difference (p>0.05) There was a statistically HIGHLY significant difference seen for the values between the groups (p<0.01) for MFI RORγt+  with higher values in AKI study arm. Ratio RORγt+:Foxp3 with higher values in AKI study arm. There was a statistically significant difference seen for the values between the groups (p<0.05) for MFI Foxp3 with higher values in CKD study arm. There was a statistically non significant difference seen for the values between the control, AKI & CKD study arms (p>0.05) for CD4%.

						95% Confidence Interval for Mean					
	Study ARM	N	Mean	Std. Deviation	Std. Error	Lower Bound	Upper Bound	Minimum	Maximum	F value	p value of one way ANOVA
CD4%	Control	10	16.033000	2.4234413	.7663594	14.299375	17.766625	11.1200	19.2300		
	AKI	40	15.448000	2.8921115	.4572830	14.523058	16.372942	10.4900	19.8300	.171	.843#
	CKD	40	15.671750	3.1271416	.4944445	14.671642	16.671858	10.0500	20.4900		
MFI FoxP3	Control	10	484.20	76.637	24.235	429.38	539.02	398	677		
	AKI	40	640.90	318.533	50.364	539.03	742.77	234	1626	3.153	.048*
	CKD	40	705.45	197.411	31.213	642.32	768.58	324	1045		
MFI RORγt^+^	Control	10	525.80	210.087	66.435	375.51	676.09	298	1015		
	AKI	40	2103.20	617.940	97.705	1905.57	2300.83	589	3028	31.899	.000**
	CKD	40	2001.65	582.696	92.132	1815.29	2188.01	1031	3231		
Ratio RORγT : FoxP3	Control	10	1.075193	.3577451	.1131289	.819278	1.331109	.6742	2.0059		
	AKI	40	4.098163	2.4077850	.3807042	3.328116	4.868210	.5741	12.8205	9.820	.000**
	CKD	40	3.154253	1.6635285	.2630269	2.622230	3.686275	1.2530	8.5251		

Regulatory T cells can co-express RORγt and Foxp3 and are shown to be both pro-inflammatory and immunosuppressive. There is a counterbalance between pathogenic (Th17) T cells that induce autoimmunity and regulatory (Foxp3+) T-cells that inhibit autoimmune tissue injury. MFI Foxp3 was higher in the CKD study arm, making it statistically significant, on comparison, with healthy volunteers and the AKI study arm. There was a statistically non-significant difference (p>0.05%) seen between healthy volunteers, AKI and CKD study arm for CD4% cells. In the post hoc analysis (Table 2) it was found that the MFI RORγt in the kidney injury group (acute and chronic) was statistically significant with respect to healthy volunteers, indicating that injury maybe attributed to T-cell dysfunction. Interestingly the ratio of RORγt:Foxp3 was also statistically significant between healthy volunteers and patients with kidney injury (acute and chronic) when analyzed individually. When comparing MFI values of RORγt and Foxp3 individually between the two kidney injury groups AKI and CKD, they were statistically insignificant and so was the ration of RORγt and Foxp3, indicating that the severity and chronicity of kidney injury may not be solely attributed to T-cell activity alone. 

**Table 2:** Post Hoc analysis of inter group, pair wise comparison of numerical outcomes of kidney disease (AKI & CKD) with RORγt+Foxp3+ Abbreviations used in Table 2: MFI = Mean fluorescence intensity; RORγt+ = Retineic-acid-receptor-related orphan nuclear receptor gamma; FoxP3 = fork-head box protein 3; AKI = Acute Kidney Injury; CKD = Chronic Kidney Disease Annotations for Table 2 : * = statistically significant difference (p<0.05) ** = statistically highly significant difference (p<0.01) # = non significant difference (p>0.05) There was a statistically HIGHLY significant difference seen for the values between the groups (p<0.01) for 1. MFI RORγt between control vs AKI and control vs CKD and 2. Ratio RORγt:Foxp3 between Control vs AKI. There was a statistically significant difference seen for the values between the groups (p<0.05) for 1. MFI Foxp3 between control vs CKD and 2. Ratio RORγt:Foxp3 between groups control vs CKD. There was a statistically non significant difference seen for the values (p>0.05) for CD4% between all the pairs - 1. MFI Foxp3 between groups Control vs AKI, AKI vs CKD, 2. MFI RORγt between AKI vs CKD and 3. Ratio RORγt:Foxp3 between AKI vs CKD.

Dependent Variable	(I) group	(J) group				95% Confidence Interval	
			Mean Difference (I-J)	Std. Error	p value	Lower Bound	Upper Bound
CD4%	Control	AKI	.5850000	1.0452735	.842#	-1.907433	3.077433
	Control	CKD	.3612500	1.0452735	.936#	-2.131183	2.853683
	AKI	CKD	-.2237500	.6610890	.939#	-1.800103	1.352603
MFI FoxP3	Control	AKI	-156.700	89.135	.190#	-369.24	55.84
	Control	CKD	-221.250^*^	89.135	.039*	-433.79	-8.71
	AKI	CKD	-64.550	56.374	.489#	-198.97	69.87
MFI RORγT	Control	AKI	-1577.400^*^	202.468	.000**	-2060.18	-1094.62
	Control	CKD	-1475.850^*^	202.468	.000**	-1958.63	-993.07
	AKI	CKD	101.550	128.052	.708#	-203.79	406.89
Ratio RORγT : FoxP3	Control	AKI	-3.0229700^*^	.6939572	.000**	-4.677697	-1.368243
	Control	CKD	-2.0790596^*^	.6939572	.010*	-3.733786	-.424333
	AKI	CKD	.9439104	.4388971	.086#	-.102631	1.990452

Since 30% of our study population had diabetes, we compared their T-cell activity with non-diabetics. Interestingly the RORγt and Foxp3 activity was statistically insignificant in diabetic patients with kidney injury in our study.

## Discussion

Despite the various advances in health care, kidney transplant remains to be the cornerstone for end stage renal disease. With the shortages in donor organs, most of these patients rely on hemodialysis, with a limited success in improving survival. Kidney injury requiring either kidney transplant or hemodialysis add to the global health and financial burden. 

In the present study, we attempted to test experimental hypothesis (Alikhan et al. 2018) [12] of the role of T-lymphocytes in kidney disease in the clinical scenario. Most of our current knowledge is based on animal (mice) models. We endeavoured to apply this literature to clinical practice by studying T-cell activity in patients with kidney injury. 

Our study population concentrated on acute and chronic kidney disease, excluding kidney injury secondary to autoimmune diseases like systemic lupus erythematosis, scleroderma or IgA nephropathy and also kidney transplantation recipients or those already receiving immuno-suppressants. Various studies suggest that the immune system is activated after kidney injury. We studied peripherally circulating T-lymphocytes in acute kidney injury and chronic kidney disease (not on maintenance hemodialysis), comparing them to healthy volunteers. Blood collection for T-cell analysis was done prior to administering any medications (example - immuno-suppressants) or before subjecting them to renal replacement therapy (dialysis/transplantation). To emphasise regarding the frequency of blood sampling, T-cell analysis was done on a one-time blood sample only. Follow-up or post-treatment analysis of T-cells was not done due to financial constraints. Patient care and management were not affected by this study.

We found that the mechanism of acute kidney injury may be explained by T-cell dysfunction - Tregs/Th17. The balance between RORγt and Foxp3 may modify the course of disease. Further studies will be needed to analyze whether these molecules can be used as markers for prognostication in treatment course. Likewise, therapies focusing on regulating the function of Tregs and inhibiting pro-inflammatory cytokines may help in reversal of acute kidney injury. AKI remains to be a major risk factor for chronic kidney disease. Similarly multiple acute hits to the kidneys in a patient with pre-existing chronic kidney disease may further decline its function. Hence timely diagnosis and intervention is crucial to reduce the eventual burden of CKD.

**Figure 4:**
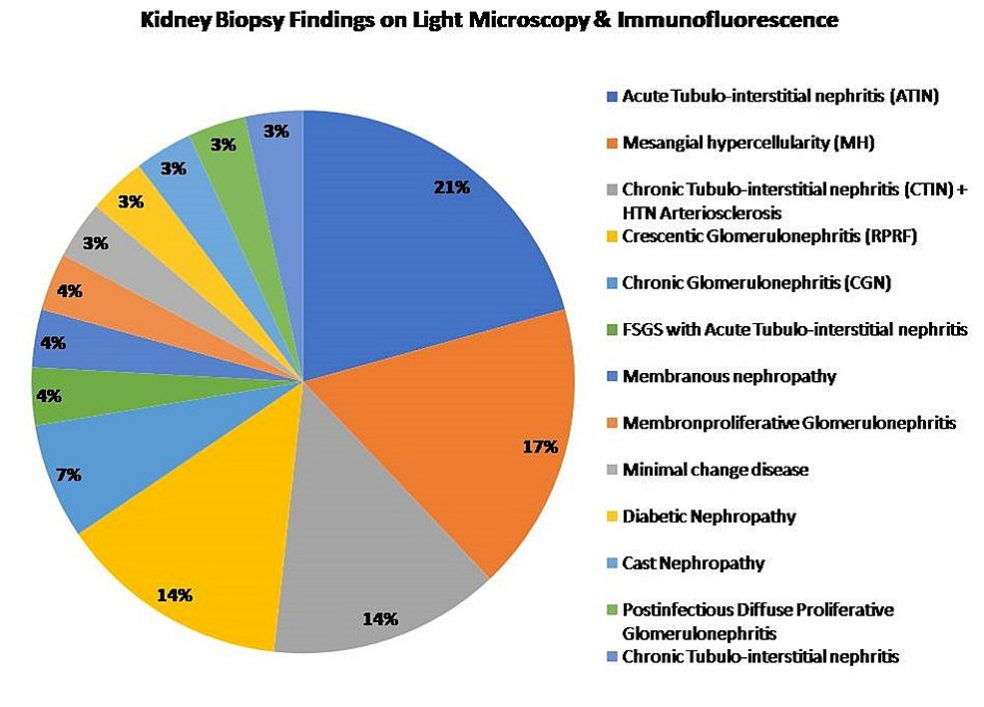
Kidney biopsy findings on light microscopy and immunofluorescence of 29 patients with kidney injury, in the study population.

Studies focusing on specific patterns of AKI would give us a clear understanding of the underlying pathological processes causing injury. In order to attribute kidney injury to T-cell dysfunction, data of 29/80 patients (Figure 4) with kidney disease were analyzed. The kidney biopsy was done, when indicated, as part of diagnostic protocol for patient management and not for the study per se. Due to financial constraints, the T-cell population in kidney biopsy was not studied. Interestingly, the ratio of RORγt:Foxp3 was found to be more than 1.5 in 24/29 patients who underwent kidney biopsy, suggesting T-cell dysfunction in both acute and chronic kidney injury. 

In an observational study by Zhu et al. in 2018 [13], it was found that compared to controls, CKD patients had an increased Th17/Treg cell ratio that was positively correlated with CKD stage. In our study, there was a statistically nonsignificant difference seen for the values between the groups (p>0.05) for CD4%, MFI Foxp3, RORγt, Ratio Rorγt:Foxp3 between the stages of CKD- G3b, G4, G5.

Limitations

Our study was limited to a small sample size. During data collection and analysis, we realized, that this study would be of great importance to clinicians if Tregs were also analysed post recovery in AKI. Just like a routine hemogram is a great tool for clinico-pathological correlation, tracking the T-cell profile of an individual in health and disease would be useful to the treating physician. Although we have computed regular follow-up of the patients with kidney injury included in this study, in terms of survival and resolution or progression of renal function, data was insufficient to correlate "one-time" test values of Treg activity (via Foxp3 or Rorγt) to predict patient outcome. Hence whether serial monitoring of Treg activity to assess prognostication of kidney injury would be valuable to know with further follow-up studies.

Future work should identify whether clinical application of quantitative or qualitative Tregs in various types of kidney disease would modify the course of disease and play a crucial role in management and reversal of kidney injury in the early stages.

## Conclusions

Our study favours the hypothesis of the role of T-cell dysfunction in kidney injury (acute and chronic kidney disease) via activity of Foxp3 and RORγt. We found that there is evidence of altered Th17/Treg activity in kidney injury, more prevalent in acute than chronic, when compared to healthy volunteers. 

We suggest studying urinary cytokines along with simultaneous analysis of T-cells in blood and on kidney biopsy to establish the role of T-cells in kidney injury. Further studies are needed to investigate the trends of T-cell activity with disease severity and progression of kidney injury in the clinical scenario.
